# Reduced frequency dosing of osimertinib in EGFR-mutant non-small cell lung carcinoma: real world data

**DOI:** 10.3332/ecancer.2024.1721

**Published:** 2024-06-28

**Authors:** Vanita Noronha, Harsh Sahu, Akhil Kapoor, Vijay Patil, Nandini Menon, Minit Shah, Dilan Davis, Rumeli Roy, Srigadha Vivek, Amit Janu, Rajiv Kaushal, Kumar Prabhash

**Affiliations:** 1 Department of Medical Oncology, Tata Memorial Hospital, Homi Bhabha National Institute (HBNI), Mumbai 400012, Maharashtra, India; 2 Department of Medical Oncology, Homi Bhabha Cancer Hospital, Varanasi 221001, Uttar Pradesh, India; 3 Department of Medical Oncology, Hinduja Hospital, Mumbai 400016, Maharashtra, India; 4 Department of Radiology, Tata Memorial Hospital, Homi Bhabha National Institute (HBNI), Mumbai 400012, Maharashtra, India; 5 Department of Pathology, Tata Memorial Hospital, Homi Bhabha National Institute (HBNI), Mumbai 400012, Maharashtra, India

**Keywords:** osimertinib, EGFR, NSCLC, low dose, financial toxicity, LMIC

## Abstract

**Introduction:**

Osimertinib is more efficacious and as safe as first-generation epidermal growth factor receptor (EGFR)-directed tyrosine kinase inhibitors. However, osimertinib is not affordable for most patients in developing nations. Moreover, the minimum biologically effective dose of osimertinib may be less than the approved dose.

**Materials and methods:**

This was a retrospective observational multicentric study aimed to describe the efficacy (objective response rate (ORR), disease control rate (DCR), progression free survival (PFS), overall survival (OS)) and toxicity of osimertinib 80 mg orally administered less frequently than daily (ranging from every other day to once-a-week) in patients with EGFR-mutated non-small cell lung cancer.

**Results:**

Between January 2021 and August 2023, we enrolled 22 patients. Six received osimertinib 80 mg once-a-week, nine received 80 mg once-in-3-days and seven received 80 mg on alternate days. Responses included 0 complete responses, 7 (31.8%) partial responses, 9 (40.9%) stable disease and 5 (22.7%) progressive disease. ORR was 31.8%, and DCR was 72.7%. Median PFS was 9.2 months (95% confidence interval (CI) 2.9–15.7), and median OS was 17.8 months (95% CI, 3.2–32.6). In patients who received reduced frequency osimertinib in the second line and beyond, the ORR was 29.4%, DCR was 70.5%, median PFS was 5.9 months (95% CI, 1.1–10.6) and median OS was 17.6 months (95% CI, 2.9–32.2). Grade 3 and higher toxicities were noted in 8 (36.3%) patients.

**Conclusion:**

Less frequent dosing of osimertinib may be a valid treatment option, especially in the second line and beyond setting in patients who cannot afford full dose daily osimertinib. This may provide an additional treatment option with a similar toxicity profile as that of standard dose osimertinib.

## Introduction

Lung cancer is one of the leading causes of cancer incidence and cancer-related deaths in India [1]. The treatment of advanced lung cancer has been revolutionised in recent years, with the identification of immunotherapy and targeted agents acting on various molecular drivers like the epidermal growth factor receptor* (EGFR)* mutations [2]. The proportion of patients with lung carcinoma that harbors *EGFR* mutation varies globally with 6% reported in African American patients, 15% in Europeans, 23% in Indians, 35% in Hispanics and 47% in Asians [3, 4].

Osimertinib is an oral, irreversible, third-generation tyrosine kinase inhibitor (TKI), that targets both *EGFR* sensitising and resistance (T790M) mutations. Osimertinib has been shown to result in a better response rate, progression-free survival (PFS) and overall survival (OS) as compared to first-generation EGFR-directed TKIs, with a similar safety profile and fewer serious adverse events [5]. It penetrates the blood-brain barrier and therefore leads to a superior response rate in the central nervous system than chemotherapy [6]. Osimertinib is also the preferred treatment as compared to pemetrexed plus platinum chemotherapy in patients whose disease has progressed on first-line EGFR-directed TKIs and have an *EGFR* T790M resistance mutation, given the better response rate, superior OS and lower rate of adverse events [7]. Even in patients whose disease has progressed on first-generation EGFR TKIs but do not have a detectable *EGFR* T790M mutation, osimertinib has been shown to be efficacious in a real world case series [8].

Although very effective, the high cost of osimertinib makes it unaffordable in India where the majority of patients do not have health insurance and pay all healthcare costs out of pocket [9, 10]. Osimertinib has been proven to not be cost effective even in high-income countries including the United States of America, Australia, Singapore and China [11, 12]. Considering the soaring healthcare costs, and the fact that determining the doses of most targeted agents like oral TKIs by the traditional phase I method of maximal tolerable dose may not be the optimal method, it is important to evaluate whether the approved dosing of various targeted drugs is appropriate [13]. Our group has recently shown that administering immunotherapy at a tenth of the approved dose is an effective strategy in patients with advanced head and neck cancers [14, 15]. Several oral TKIs like ceritinib, and oral hormonal drugs like abiraterone have been shown to be effective at a fraction of the approved doses.

Osimertinib has a half-life of 59 hours, a high volume of distribution, minimal first pass metabolism and low clearance [16]. In the AURA1 study, the objective response rate (ORR) to osimertinib was similar across the dose levels ranging from 20 to 240 mg (20 mg – 52%, 40 mg – 43%, 80 mg – 52%, 160 – 58%, 240 mg – 52%), with similar serious adverse events occurring at all the dose levels [17]. Our group had earlier demonstrated that in an orthotopic mouse model, administering osimertinib once-a-week was as effective as administering it daily in preventing the homing of adenocarcinoma PC9 luciferase cells to the mice’s lungs [18]. In a recent case series of 29 patients with non-small cell lung cancer (NSCLC) with disease progression on a first-generation EGFR-directed oral TKI (over 75% had also received cytotoxic chemotherapy) and were noted to harbor *EGFR* T790M mutation at progression, 13 (44.8%) patients received osimertinib 40 mg orally daily (dose reduced for adverse effects), while the rest were treated with standard dose osimertinib. The response rate and PFS were similar between the cohort of patients who received full dose osimertinib (62.5% and 3.46 months, respectively) compared to those who received osimertinib 40 mg orally daily (76.9% and 5.4 months, respectively); *p* = 0.56 [19]. At our center, osimertinib is only available as 80 mg tablets, thus prescribing a lower dose is a challenge. A case report described a Chinese gentleman with metastatic lung adenocarcinoma with *EGFR* exon 19 deletion whose disease had progressed on afatinib, and whose blood *EGFR* T790M was negative. He received osimertinib 80 mg orally every other day, with a good partial response (PR) and a PFS of 9 months [20]. Based on the encouraging preclinical and early clinical data, we administered osimertinib 80 mg orally at a reduced frequency on a compassionate basis to patients with *EGFR* mutant lung cancer who were unable to afford full dose osimertinib, refused or had progressed on intravenous chemotherapy and did not have any other treatment options available. We aimed to explore the efficacy of osimertinib administered at a reduced frequency.

## Materials and methods

### General study details

This was a retrospective, observational multicentric study conducted at two centers of the Tata Memorial Center (TMC): Tata Memorial Hospital (Mumbai, Maharashtra) and Homi Bhabha Cancer Hospital and Mahamana Pandit Madan Mohan Malaviya Cancer Center (Varanasi, Uttar Pradesh). Both hospitals are academic tertiary care centers in India. The study was approved by the Institutional Ethics Committee (IEC) of TMC. As this was a retrospective analysis of electronic medical records, the IEC waived the requirement to obtain written informed consent. The study was conducted according to the principles laid down by the International Conference on Harmonization Good Clinical Practice guidelines, the Declaration of Helsinki, and Schedule Y (Drugs and Cosmetics Act, 1940) and the guidelines established by the Indian Council of Medical Research. Since it was a retrospective study, it was not registered in a publicly accessible clinical trials registry. No funding was received for the study.

### Aims and objectives

The primary objective was to determine the PFS of patients who were treated with reduced frequency osimertinib. Secondary objectives included determination of the ORR, disease control rate (DCR), toxicity, OS and pattern of progression.

### Study population

We included patients aged 18 years or older, with histologically proven NSCLC harboring *EGFR* mutation who received osimertinib 80 mg less frequently than once daily. There were no specific exclusion criteria.

### Methodology

We searched the electronic medical records and the database maintained in the Department of Medical Oncology to identify the patients who received osimertinib at reduced frequency. We recorded the clinicodemographic, histopathologic and molecular testing and therapy details. Patients had been prescribed osimertinib 80 mg orally either once-a-week, once-in-3-days or on alternate days, at the discretion of the prescribing oncologist. The reasons for prescribing osimertinib at reduced frequency were recorded. Response assessment was based on the response evaluation criteria in solid tumours, version 1.1. Responses were categorised as complete response (CR), PR, stable disease (SD) or progressive disease (PD). CR was defined as the disappearance of all target lesions; any pathological lymph nodes reduced to less than 1 cm in the short axis). PR was defined as a reduction of at least 30% in the sum of the longest diameters of the target lesions, taking the sum of the longest diameters at baseline as the reference. PD was defined as an increase of at least 20% in the sum of the longest diameters of the target lesions as compared to the smallest sum, provided the sum had also increased by at least 5 mm. The appearance of one or more new lesions was also considered PD. SD was defined as a change in the sum of the longest diameters between −30% and +20%; insufficient reduction to qualify for PR, and an insufficient increase to qualify for PD. ORR was calculated as the proportion of patients who had CR and PR; DCR was defined as the proportion of patients who had CR, PR and SD. PFS was defined as the duration between the start of reduced frequency osimertinib and disease progression or death due to any cause. OS was defined as the duration between the initiation of reduced frequency osimertinib and death due to any cause; patients who were alive were censored on the date of last follow up. Toxicities were graded according to the Common Terminology Criteria for Adverse Events (CTCAE), version 5.0. The worst toxicities experienced while the patients were on reduced frequency osimertinib were recorded.

### Statistics

We did not calculate a sample size *a priori* as this was a retrospective study. Data analysis was done using the Statistical Package for the Social Sciences (IBM Corp. Released 2011. IBM SPSS Statistics for Windows, Version 20.0. Armonk, NY: IBM Corp.), and R Studio, version 4.1. Baseline characteristics, ORR, DCR and toxicities have been presented with descriptive statistics. Non-continuous variables have been expressed in absolute numbers and percentages, and continuous variables as medians with ranges. The Kaplan–Meier method was used to estimate the PFS and OS.

## Results

Between January 2021 and August 2023, we identified 22 patients who had received reduced frequency osimertinib. Six (27.3%) patients received 80 mg once-a-week, nine (40.9%) patients received 80 mg once-in-3-days and seven (31.8%) received 80 mg on alternate days. There were 21 (95.5%) patients with metastatic disease; one (4.5%) had stage III disease; all patients were receiving therapy with palliative intent. The reasons for prescribing reduced frequency osimertinib rather than the standard dose were financial in 20 (90.9%) patients and toxicity from standard dose osimertinib (grade 3 thrombocytopenia) in 2 (9.1%) patients. *EGFR* T790M mutation was present in 15 (68.2%) patients. Reduced frequency osimertinib was used in the first line in 5 (22.7%) patients. Among the 17 (77.2%) patients who received reduced frequency osimertinib in the second line and beyond setting, *EGFR* T790M mutation was present in 14 (82.4%) patients. Baseline characteristics have been described in Table 1.

**Figure 1.**
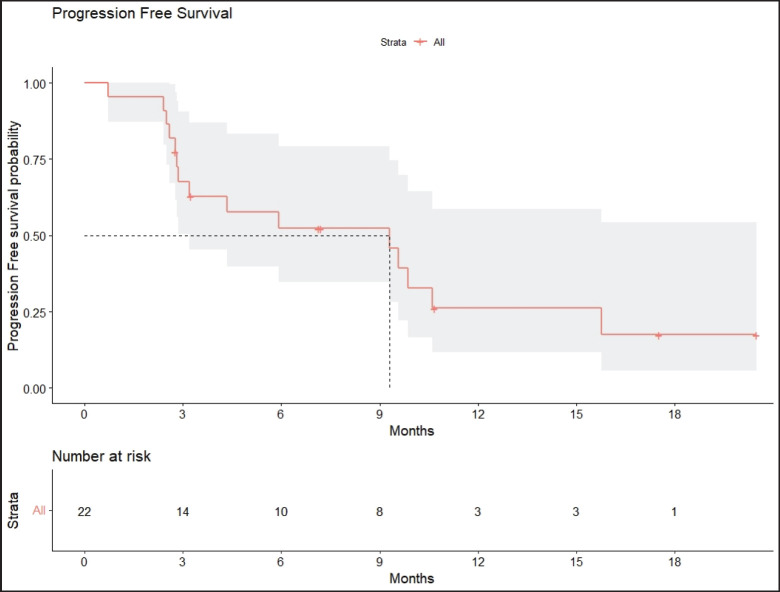
PFS of patients with *EGFR* mutant NSCLC who were treated with osimertinib 80 mg at a frequency less than once daily.

**Figure 2.**
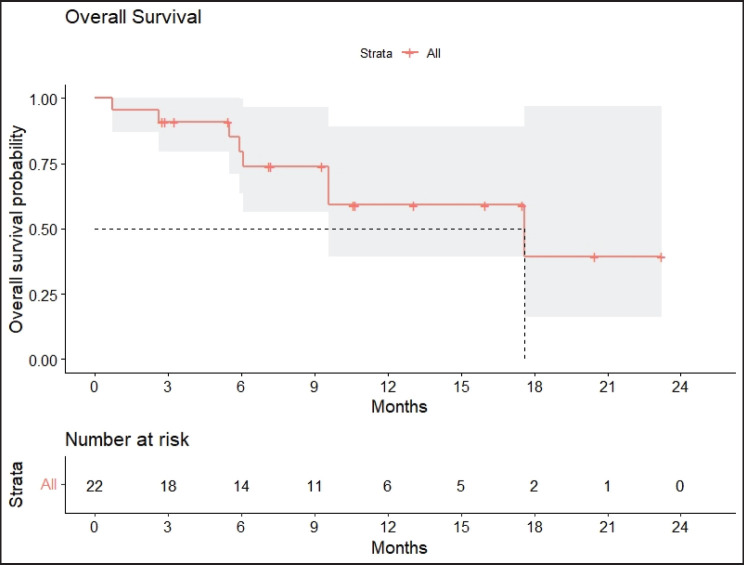
OS of patients with *EGFR* mutant NSCLC who were treated with osimertinib 80 mg at a frequency less than once daily.

**Figure 3.**
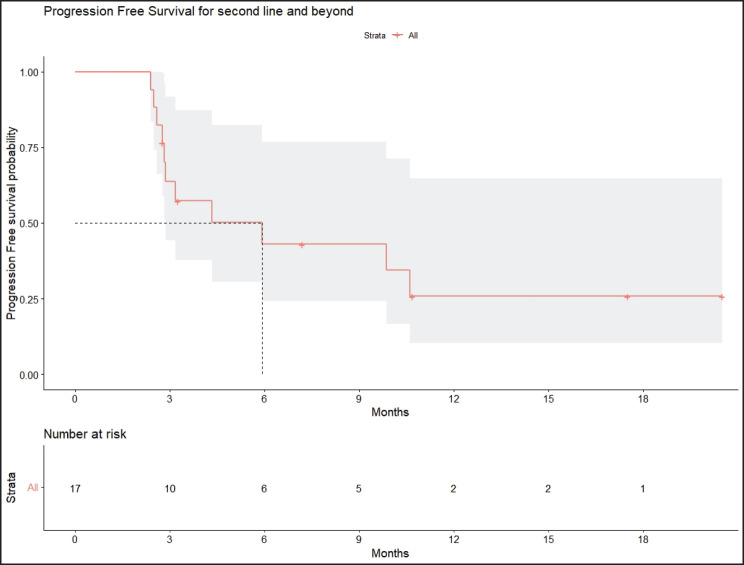
PFS of patients with *EGFR* mutant NSCLC who were treated with osimertinib 80 mg at a frequency less than once daily in the second line and beyond setting.

**Figure 4.**
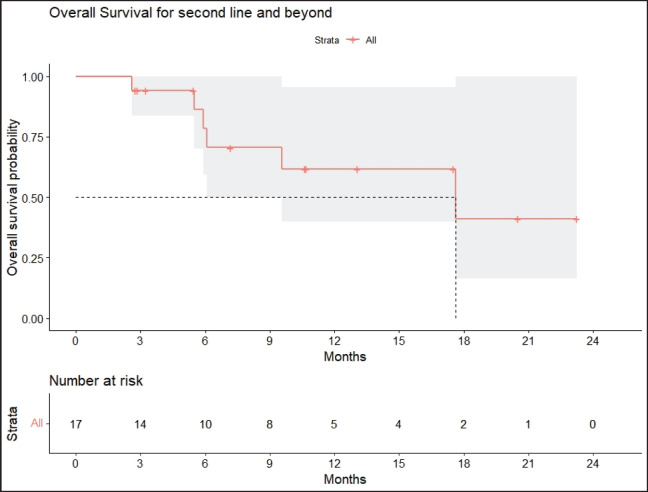
OS of patients with *EGFR* mutant NSCLC who were treated with osimertinib 80 mg at a frequency less than once daily in the second line and beyond setting.

**Figure 5.**
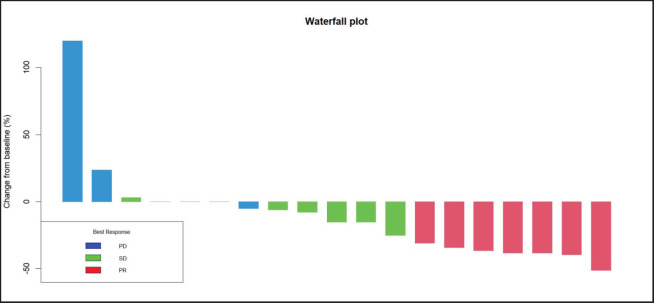
Waterfall plot of the responses of patients with *EGFR* mutant NSCLC who were treated with osimertinib 80 mg at a frequency less than once daily.

**Figure 6.**
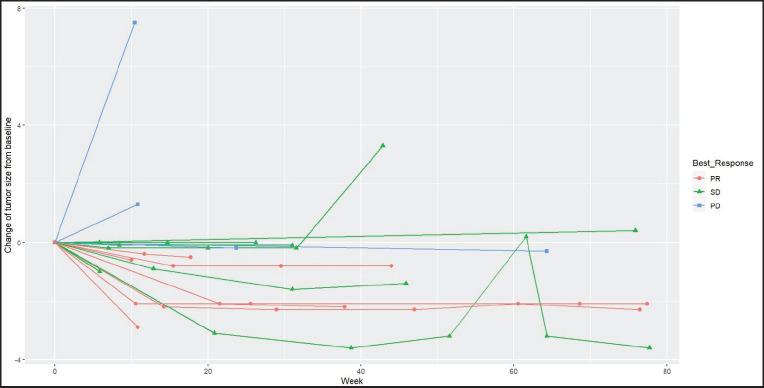
Spider plot of the responses of patients with *EGFR* mutant NSCLC who were treated with osimertinib 80 mg at a frequency less than once daily. (PR=partial response, SD=stable disease, PD=progressive disease)

Radiologic response assessment was performed in 21 (95.5%) patients. The ORR was 33.3% (7/21), and the DCR was 76.2% (16/21). Responses comprised no patients with CR, 7 (33.3%) with PR, 9 (42.9%) with SD and 5 (23.8%) with PD. The median PFS was 9.2 months (95% confidence interval (CI), 2.9–15.7) (Figure 1). The median OS was 17.8 months (95% CI, 3.2–32.6) (Figure 2). When used in the second line and beyond setting, the median PFS was 5.9 months (95% CI, 1.1–10.6) (Figure 3), and the median OS was 17.6 months (95% CI, 2.9–32.2) (Figure 4). Radiologic responses in the cohort of 17 patients who received reduced frequency osimertinib in the second line and beyond setting included 5 (29.4%) PR, 7 (41.2%) SD and 5 (29.4%) PD; thus, the ORR was 29.4% and the DCR was 70.5% in the second line and beyond setting. Figures 5 and 6 show the percentage changes in the tumour burdens of individual patients. The ORR noted with the use of osimertinib at the various dose levels were as follows: alternate day dosing = 33.3% (2/6), once-in-3-days = 22.2% (2/9) and once-a-week dosing = 50% (3/6).

The lung was the most common site of disease progression, with progression occurring in the lung primary in 4 (18.2%) patients and as metastatic lung nodules in 3 (13.6%) patients. Progression in the brain developed in 2 (9.1%) patients. The sites of disease progression on reduced frequency osimertinib are provided in Table 2.

Table 3 shows the toxicities associated with low-dose osimertinib. Anemia and thrombocytopenia were the most common toxicities. Grade 3 and higher toxicities were noted in 8 (36.3%) patients. Adverse events requiring dose interruptions occurred in 6 (27.3%) patients, and included thrombocytopenia in 4 (18.2%), elevated transaminases in 1 (4.5%) patient and pneumonitis in 1 (4.5%) patient. Thrombocytopenia and transaminase elevation resolved after dose interruption in all patients. One patient developed fatal interstitial pneumonitis that was suspected to be caused by osimertinib.

Two (9%) patients were changed from standard dose osimertinib, i.e., 80 mg once daily to reduced frequency osimertinib due to grade 3 thrombocytopenia following the standard dosing. Both these patients had grade 3 thrombocytopenia even with alternate day dosing. In the first patient, the frequency of osimertinib administration was decreased to every other day in the first month of starting osimertinib 80 mg once daily, while the frequency was decreased in the second month in the second patient. Both these patients had SD as the best response to reduced frequency osimertinib. One of them died due to disease progression after 9 months of treatment with reduced frequency osimertinib, while the second patient continues on therapy following 9 months on reduced frequency osimertinib. In one patient who had disease progression on treatment with osimertinib once-a-week, the dose of osimertinib was increased, initially to a dose of 80 mg on alternate days on which she progressed after 2 months, and subsequently to the standard 80 mg once daily dose, which also resulted in disease progression after 2 months.

## Discussion

Osimertinib is the treatment of choice for NSCLC with *EGFR* sensitising mutations as well as T790M resistance mutations. However, due to its high cost, most patients in developing countries like India are unable to receive osimertinib, particularly at the approved dose of 80 mg orally once daily. In our real-world cohort, 90.2% of patients received reduced frequency osimertinib due to their inability to afford the standard dose regimen. Of the patients in our cohort, 77.2% had already received alternative therapies including chemotherapy, the combination of chemotherapy + gefitinib, or a first-generation EGFR-directed oral TKI. Administering osimertinib 80 mg at less than daily frequency led to an ORR of 33.3%, median PFS of 9.2 months (95% CI, 2.9–15.7) and a median OS of 17.8 months (95% CI, 3.2–32.6). Considering that these patients had essentially no other treatment options, this efficacy appears to be promising, and worthy of evaluation in a randomised trial.

The landmark FLAURA study reported that treatment with osimertinib in the first line resulted in a median PFS of 18.9 months (95% CI, 15.2–21.4) and a median OS of 35.8 months (95% CI, 34.5–41.8) [5]. In a retrospective study of 524 patients who received osimertinib in the salvage setting, the ORR was 55.6%, DCR was 88.9%, median PFS was 17.2 months and median OS was 20 months (95% CI: 15.1–24.9) [21]. In patients who are unable to receive osimertinib, alternative treatment options include first or second-generation TKIs, chemotherapy or the combination of an EGFR-directed TKI with chemotherapy, provided the patient has not already received these treatments. Therapy with gefitinib in the first line led to an ORR of 55%, median PFS of 9.2 months and median OS of 17.5 months [22]. In a study conducted by our group, the combination of pemetrexed and platinum therapy in the first line led to an ORR of 45.3%, DCR of 78.6%, median PFS of 5.6 months (95% CI, 4.2–7.0) and median OS of 22.6 months (95% CI, 18.6–26.6) [23]. Another study conducted by us reported that the combination of chemotherapy with gefitinib resulted in an ORR of 75.3%, DCR of 87.9%, median PFS of 16 months (95% CI, 13.5–18.5) and median OS of 27.5 months (95% CI, 24.8–30.8) [24, 25]. In the current study, the efficacy of reduced frequency osimertinib (ORR = 33.3%, DCR = 72.7%, median PFS = 9.2 months, median OS = 17.8 months) appeared to be comparable to that of a first-generation EGFR-directed TKI in the first line setting. However, an important point to note is that our cohort included only 5 (22.7%) patients who received reduced frequency osimertinib in the first line setting; the remaining 17 (77.2%) patients were treated in the second line and beyond setting. Thus, our study cannot answer whether reduced frequency osimertinib is a valid first line therapeutic option over a first-generation EGFR-directed TKI in patients for whom standard dose osimertinib is not feasible.

In the AURA3 trial, in which osimertinib was compared to pemetrexed + platinum chemotherapy in the second line setting (following progression on a prior EGFR-directed oral TKI) in patients with *EGFR* T790M mutation, the ORR was 71%, median PFS was 10.1 months (95% CI, 8.3–12.13) and median OS was 26.8 months (95% CI, 23.5–31.5) [6]. The corresponding efficacy parameters noted in the patients who received chemotherapy in the AURA3 trial were an ORR of 31%, median PFS of 4.4 months (95% CI, 4.2–5.6) and median OS of 22.5 months (95% CI, 20.2, 28.8). A recent retrospective European study in 135 patients reported that chemotherapy after progression on first-line TKI resulted in a median PFS of 5.4 months (95% CI, 4.7–6.1), and median OS of 15.3 months (95% CI, 116–18.9) [26]. In an earlier real world study report, we found that osimertinib at 80 mg a day in the second line and beyond setting for 17 patients with *EGFR* T790M mutation led to an ORR of 55% and DCR of 90.9%; survival data were immature at the time of this report [27]. In our present study, 82.4% of the patients who received reduced frequency osimertinib had *EGFR* T790M mutation in the second line and beyond setting; therapy resulted in an ORR of 29.4%, DCR of 70.5%, median PFS of 5.9 months and median OS of 17.6 months. These outcomes appeared to be inferior to those resulting from standard dose osimertinib, and comparable to those from chemotherapy. Similar to the observations from the AURA1 study, in which the ORR was similar across multiple dose levels from 20 mg orally daily to 240 mg orally daily, we found that the ORR did not appear to increase with an increase in the frequency of osimertinib administration (once-a-week dosing = 50%, once-in-3-days = 22.2% and alternate day dosing = 33.3%), with the caveat that the number of patients treated at each dose frequency cohort was too small to draw any definitive conclusions [17]. Osimertinib is orally administered, requires less hospital visits and has lower toxicity than chemotherapy. Thus, reduced frequency or low dose osimertinib may be a valid option in patients with *EGFR* mutant NSCLC who cannot afford or tolerate full dose osimertinib. This provides an additional line of therapy. Reduced frequency or low dose osimertinib may also be useful in patients who cannot afford full dose osimertinib, have progressed on first- or second-generation EGFR TKIs and are unfit for or refuse intravenous chemotherapy.

A quarter of the patients who received osimertinib 80 mg orally daily as first-line therapy in the FLAURA study required dose interruptions due to QT prolongation, reduced appetite, diarrhea and pneumonia [5]. In the AURA3 trial, 14% of patients treated with osimertinib needed dose interruptions due to adverse events [6]. In our study, reduced frequency osimertinib was interrupted in 6 (27.2%) patients due to thrombocytopenia, transaminase elevation and pneumonia. In the AURA3 trial, any grade thrombocytopenia developed in 10% of patients and grade 3 thrombocytopenia in <1% of patients [6].

In our study, reduced frequency osimertinib led to thrombocytopenia in 12 (54.5%) patients; grade 1 in 5 (22.7%), grade 2 in 3 (13.6%), grade 3 in 3 (13.6%) and grade 4 in 1 (4.5%) patient. Although this level of thrombocytopenia appears high, this was probably a spurious observation, due to selection bias. Two patients in our cohort had earlier developed thrombocytopenia on full dose daily osimertinib, following which they were changed to reduced frequency osimertinib. These patients developed recurrent thrombocytopenia on reduced frequency osimertinib as well, which probably reflected an increased propensity to this toxicity in these patients. Additionally, most of the patients in our cohort received osimertinib at a reduced frequency on compassionate basis as they did not have any other treatment options available. Given their pretreated status, the cohort may have represented a less fit group of patients who were at a higher risk of toxicities. Grade 3 transaminase elevation has been reported to occur in only 1% of patients treated with standard dose osimertinib [7, 8]. In our study, grade 3 transaminase elevation was seen in 1 (4.5%) patient. Interstitial lung disease has been reported in 4% of patients treated with standard dose osimertinib. In our study, 1 (4.5%) patient who was on alternate day osimertinib died of suspected osimertinib-induced lung toxicity. Thus, reduced frequency or low dose osimertinib may not be safer than standard dose osimertinib, and similar careful monitoring for toxicity is recommended.

Our study was limited by the retrospective observational nature of the analysis and the small sample size. Our study cohort was heterogenous and included patients who received reduced frequency osimertinib in various treatment lines and at varying frequencies. Further studies with larger sample sizes and prospective studies will be required to definitively establish the utility of reduced frequency or lower doses of osimertinib. The optimal dosing regimen of osimertinib needs to be established in a well-conducted study.

## Conclusion

Reduced frequency dosing of osimertinib may be a valid treatment option, especially in the second-line setting in patients who cannot afford standard dose osimertinib. This may provide an additional treatment option in such patients and may result in a similar toxicity profile as compared to the standard dose osimertinib.

## Conflicts of interest

There are no conflicts of interest.

## Funding

Nil.

## Artificial intelligence used

No.

## Presented elsewhere

No.

## Author contributions

**Vanita Noronha** guarantor of integrity of the entire study, study concept and design, data analysis, manuscript preparation; **Harsh Sahu** guarantor of integrity of the entire study, data analysis, manuscript preparation; **Akhil Kapoor** study concept and design, literature search, manuscript editing; **Vijay Patil** data analysis, manuscript editing; **Nandini Menon** literature search, manuscript editing; **Minit Shah** study concept and design, manuscript editing; **Dilan Davis M** data analysis, manuscript preparation; **Rumeli Roy** manuscript editing; **Srigadha Vivek** manuscript preparation,** Amit Janu** manuscript editing;** Rajiv Kaushal** manuscript editing; **Kumar Prabhash** guarantor of integrity of the entire study, study concept and design, data analysis, manuscript preparation, manuscript editing.

## Figures and Tables

**Table 1.** Baseline demographic and disease-related characteristics of patients with *EGFR* mutant NSCLC who were treated with reduced frequency osimertinib.

Baseline characteristics	Number of patients (%) *N* = 22
Age in years (Mean ± SD)	54.6 ± 10.9
Male sex	9 (40.9)
Histology	
Adenocarcinoma	20 (90.0)
Squamous carcinoma	1 (4.5)
Adenosquamous carcinoma	1 (4.5)
Disease stage	
Stage III^a^	1 (4.5)
Stage IV	21 (95.5)
Site of metastasis	
Lung	13 (59.1)
Non-regional lymph node	19 (86.4)
Pleural effusion	6 (27.3)
Pleural nodule	6 (27.3)
Bone	12 (54.5)
Liver	6 (27.3)
Adrenal	1 (4.5)
Soft tissue	1 (4.5)
Brain	10 (45.5)
Leptomeninges	1 (4.5)
Line of therapy in which reduced frequency osimertinib was administered	
First line	5 (22.7)
Second line and beyond	17 (77.2)
Second line	9 (40.9)
Third line	4 (18.2)
Fourth line	2 (9)
Fifth line	1 (4.5)
Sixth line	1 (4.5)
Previous treatment	
Gefitinib alone	9 (40.9)
Gefitinib + pemetrexed + carboplatin	4 (18.2)
Pemetrexed + carboplatin	6 (27.3)
Gemcitabine	3 (13.6)
Taxane (docetaxel/paclitaxel)	4 (18.2)
Paclitaxel + carboplatin	1 (4.5)
Gefitinib + bevacizumab	3 (13.6)
Crizotinib	1 (4.5)
Nivolumab	2 (9.1)
Ipilimumab	1 (4.5)
Whole brain radiotherapy	7 (31.8)
Type of *EFGR* alteration	
Exon 19 deletion	17 (77.3)
Exon 21 L858R mutation	4 (18.2)
Exon 18 G719X	1 (4.5)
Exon 20 T790M	15 (68.2)
*EGFR* amplification	2 (9.1)
Next generation sequencing (NGS) done	13 (56.5)
Other concurrent mutations noted in the NGS	
*TP53* mutation	4 (18.2)
*EFGR* exon 10 deletion	1 (4.5)
*CDK4* amplification	1 (4.5)
*PIK3CA* mutation	3 (13.6)
* KRAS* mutation	1 (4.5)
*FGFR4* mutation	1 (4.5)

SD = Standard deviation; ^a^This patient had a right upper lobe lung mass with a left supraclavicular lymph node involvement. The disease could not be encompassed in a single radiation portal, hence palliative intent therapy was administered. The patient received gefitinib with bevacizumab in the first line setting; at progression, repeat biopsy revealed *EGFR* T790M mutation and was started on osimertinib 80 mg orally once-a-week

**Table 2.** Sites of progression in patients with *EGFR* mutant NSCLC following therapy with reduced frequency osimertinib.

Site of progression	Number of patients (%) *N* = 22
Lung primary	4 (18.2)
Lung metastasis	3 (13.6)
Lymph node	1 (4.5)
Pleural effusion	2 (9.1)
Pleural nodule	2 (9.1)
Bone	1 (4.5)
Adrenal	1 (4.5)
Brain	2 (9.1)
Leptomeninges	1 (4.5)

**Table 3.** Worst grade adverse events noted in patients with *EGFR* mutant NSCLC who were treated with reduced frequency osimertinib. Toxicity grading was as per the CTCAE, version 5.0.

Type of adverse event	Number (%) of patients who experienced adverse events *N* = 22				
	Grade 1	Grade 2	Grade 3	Grade 4	Grade 5
Nausea/vomiting	1 (4.5)	0	0	0	0
Fatigue	1 (4.5)	0	0	0	0
Constipation	1 (4.5)	0	0	0	0
Anemia	9 (40.9)	4 (18.2)	0	0	0
Thrombocytopenia	5 (22.7)	3 (13.6)	3 (13.6)	1 (4.5)	0
Transaminitis	2 (9.1)	1 (4.5)	0	0	0
Hyponatremia	2 (9.1)	3(13.6)	2 (9.1)	1 (4.5)	0
Renal dysfunction	0	1 (4.5)	0	0	0
Pneumonitis	0	0	0	0	1 (4.5)
